# Small Interfering RNA for Gliomas Treatment: Overcoming Hurdles in Delivery

**DOI:** 10.3389/fcell.2022.824299

**Published:** 2022-07-08

**Authors:** Xin-Qi Teng, Jian Qu, Guo-Hua Li, Hai-Hui Zhuang, Qiang Qu

**Affiliations:** ^1^ Department of Pharmacy, Xiangya Hospital, Central South University, Changsha, China; ^2^ National Clinical Research Center for Geriatric Disorders, Xiangya Hospital, Central South University, Changsha, China; ^3^ Department of Pharmacy, The Second Xiangya Hospital, Institute of Clinical Pharmacy, Central South University, Changsha, China

**Keywords:** gliomas, glioblastoma, siRNA, delivery, nanoparticles, therapy-resistance

## Abstract

Gliomas are central nervous system tumors originating from glial cells, whose incidence and mortality rise in coming years. The current treatment of gliomas is surgery combined with chemotherapy or radiotherapy. However, developing therapeutic resistance is one of the significant challenges. Recent research suggested that small interfering RNA (siRNA) has excellent potential as a therapeutic to silence genes that are significantly involved in the manipulation of gliomas’ malignant phenotypes, including proliferation, invasion, metastasis, therapy resistance, and immune escape. However, it is challenging to deliver the naked siRNA to the action site in the cells of target tissues. Therefore, it is urgent to develop delivery strategies to transport siRNA to achieve the optimal silencing effect of the target gene. However, there is no systematic discussion about siRNAs’ clinical potential and delivery strategies in gliomas. This review mainly discusses siRNAs’ delivery strategies, especially nanotechnology-based delivery systems, as a potential glioma therapy. Moreover, we envisage the future orientation and challenges in translating these findings into clinical applications.

## Introduction

Gliomas are the most common internal tumors of the central nervous system (CNS), whose global annual incidence is about 6 per 100,000 individuals. Men are 1.6-fold more likely to be diagnosed than women (Wesseling and Capper, 2018; Ostrom et al., 2019). Glioblastoma (GBM) is the highest grade (Grade IV) glioma in WHO’s updated classification of astrocytic tumors. Surgery is safely feasible, followed by concomitant postoperative radiotherapy, combined with adjuvant temozolomide (TMZ) chemotherapy, which constitutes the standard treatment for newly diagnosed adult GBM patients (Messaoudi et al., 2015; Weller et al., 2017). However, this cancer’s aggressiveness and drug resistance lead to a high recurrence and low average survival times (Ding et al., 2020). Therefore, it is necessary to provide more effective treatment methods, such as precisely targeted therapy, to design effective targeted agents for specific genes that cause the GBM development. Genetic tools have been developed, including RNA interference (RNAi), for silencing genes related to cancer progression.

RNAi refers to a phenomenon that is highly conserved in the evolutionary process. Nowadays, RNAi has become a gene-silencing tool that uses exogenous small interfering RNA (siRNA), short hairpin RNA (shRNA), and micro RNA (miRNA) to inhibit the expression of many genes (Khatri et al., 2012). All these RNAs are cleaved by an enzyme called Dicer, which uses distinct modules for recognizing dsRNA termini (Bernstein et al., 2001; Sinha et al., 2018). Among them, siRNA is the most commonly used structure and is the focus of our review. The research on miRNA drugs is much less than on siRNA drugs, mainly due to their mechanism of action. Compared to exogenous siRNA, the action mechanism of endogenous miRNAs is more complex because they often act on hundreds of genes and are challenging to silence specific genes, resulting in unexpected side effects (Zhang et al., 2021). siRNAs have great potential to target any cancer protein and have been widely used to explore gene function and treat infectious diseases and malignant tumors (Li et al., 2005; Song et al., 2018; Cunha-Santos et al., 2020).

siRNAs offer a novel and beneficial strategy for glioma therapy. When delivered to cancer cells, they could silence target genes associated with diverse gliomas’ hallmarks. Studies have shown that siRNA can silence carcinogenic or promoting genes (Wei et al., 2021) and hypoxia-related factors (Grassi et al., 2020), as well as regulate cancer stem cells (Liang et al., 2020) to effectively inhibit the malignancy of gliomas, which are potential therapeutic targets for gliomas treatment. Moreover, siRNA has shown great promise in potentiating chemotherapy by sensitizing drug-resistant cancer cells. Chemotherapy drugs used for malignant glioma treatment often fail due to drug resistance. Gene therapies using siRNA to knockdown specific genes such as RECQL4 or MTCH2 have shown great promise in potentiating chemotherapy by sensitizing resistant cancer cells (Król et al., 2020; Yuan et al., 2021). Likewise, the therapeutic siRNA was also demonstrated to improve GBM cells’ radio-sensitivity (Kievit et al., 2017). Immunotherapy is a promising treatment method for tumors, and satisfactory results have been achieved in some patients. However, more patients have no or little clinical benefit due to immune evasion (Binnewies et al., 2018). siRNAs have been reported to target different molecules on the surface of immune cells and tumor cells, including programmed cell death ligand-1 (PD-1) and its ligand PD-L1, as well as STAT3, which participate in the process of immune activation, to lessen tumor immune evasion and improve anti-tumor immunity (Nduom et al., 2016).

Recent research on siRNA drugs has proved their clinical therapeutic potential in various diseases. For instance, Lumasiran, a newly developed siRNA drug used to treat primary type 1 hyperoxaluria, has a superior therapeutic effect (Garrelfs et al., 2021). However, there are many barriers to the clinical application of siRNA for gliomas, including difficulty in penetrating the blood-brain barrier (BBB), poor stability, short blood circulation, extracellular and intracellular barriers, off-target effects, interferon response, tumor accumulation, and low-efficiency intracellular release of siRNA (Chen et al., 2018). Therefore, the key to achieving siRNA-based therapeutics’ optimal gene silencing effect is safe and effective delivery strategies. With the significant development of delivery strategies, siRNA therapy offers increasing prospects for glioma treatment (Kumthekar et al., 2021) (Fu et al., 2021). For example, Polo-like kinase 1 (PLK1) was found highly expressed in GBM, and PLK1 inhibition was reported to induce mitotic catastrophe, G2/M cell cycle arrest, and DNA damage, leading to caspase-mediated apoptosis in GBM cells (Higuchi et al., 2018). Recently, an Angiopep-2 functionalized siRNA nanocapsule has been designed to deliver PLK1 siRNA in GBM. Inject it intravenously into nude mice bearing orthotopic U87MG-luc human GBM tumor has a satisfactory therapeutic effect (Zou et al., 2020).

There are a lot of articles on siRNA research that have been published in the recent past, and growing evidence shows that siRNA therapy is worth investigating direction for many diseases. This review focuses on the past five years’ efforts to use various delivery strategies of siRNA for glioma treatment. We also highlight the promising strategies concerning target selection and combination therapies.

## The Mechanism of RNA Interference and Small Interfering RNA for Target Messenger RNA Inhibition

Gene therapy is a promising way to tackle different malignancies to modulate particular gene expression patterns in tumor pathogenesis (Altanerova et al., 2019). siRNA therapy is a type of gene therapy that has been widely used in clinical and experimental treatments for cancers due to its effective and selective inhibition of gene expression (Grzelinski et al., 2006).

**FIGURE 1:**
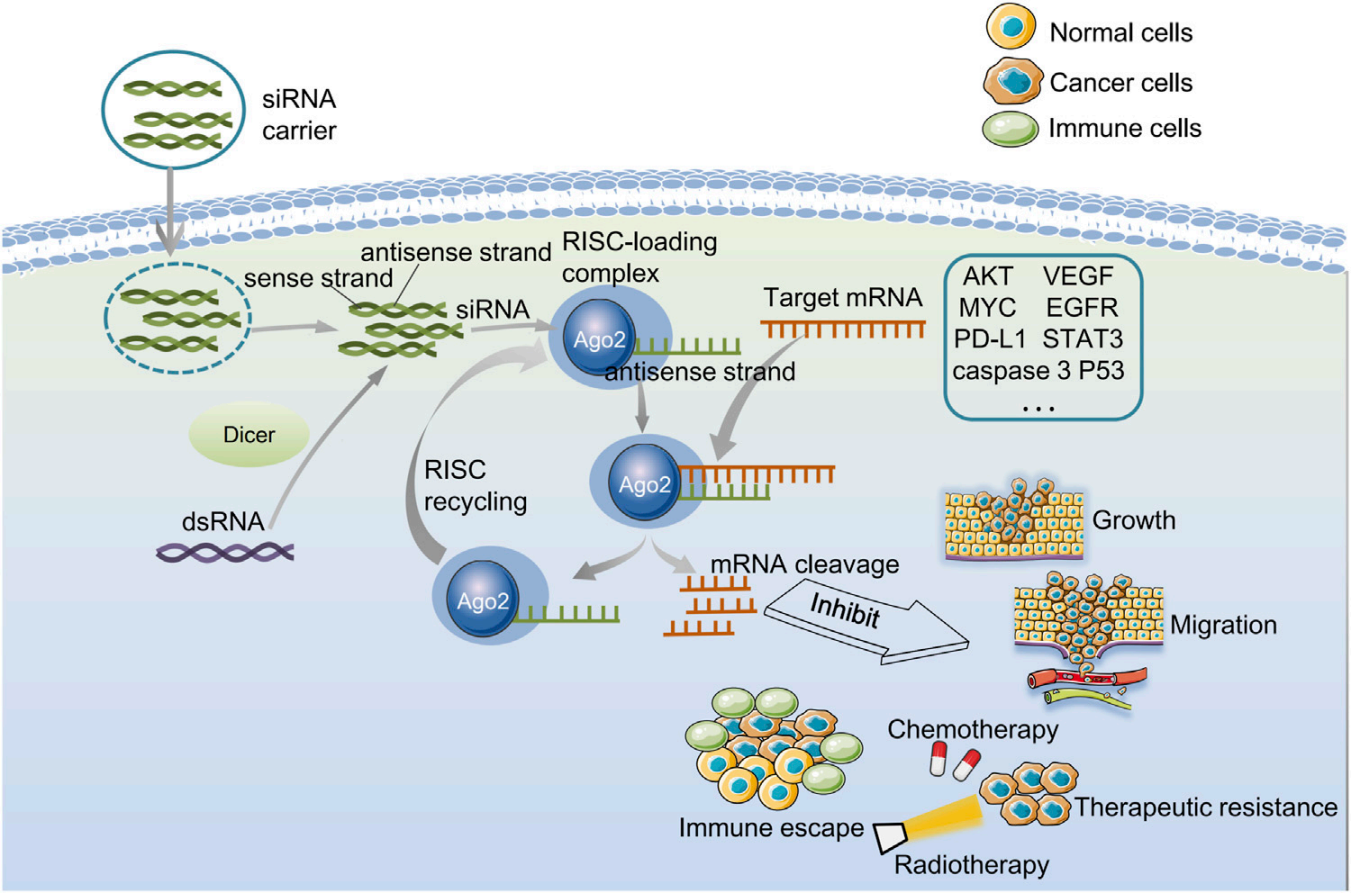
Biological characterization of gene silenced by endogenous siRNA and exogenous siRNA. Dicer cleaves the dsRNA for the endogenous pathway and gives rise to siRNAs. Then siRNA molecule is incorporated into an RNA-induced silencing complex (RISC) in the cell cytoplasm, and the sense strand is cleaved by Argonaute-2. Next, the antisense strand of the activated RISC binds to and cleaves the target mRNA that is complementary to the antisense strand, preventing translation and thereby silencing gene expression. The activated RISC can be recycled, after several cycles of synthesis-cutting, the role of RNAi is continuously amplified, and finally, the target mRNA is completely degraded. For exogenous pathways, siRNA-carriers deliver siRNAs to glioma cells, and siRNA can be directly loaded into RISC without pre-treatment with the dicer enzyme. siRNA can silence cancer-related genes, thereby inhibiting cell growth and migration, reducing drug resistance, and improving anti-tumor immunity.

RNAi was first discovered in the 1990s (Fire et al., 1998). It is initiated by double-stranded RNA (dsRNA) targets endogenous cells with transposable factors in viruses and genomes (Ullu et al., 2002). Dicer cleaves the dsRNA into 21 nucleotides, double-stranded fragments composed of a sense and an antisense strand, namely siRNA. This cleavage leads to the overhang of two nucleotides at the 3′ends and monophosphate at the 5′ends (Hamilton and Baulcombe, 1999). Then siRNA migrates to the RNA-induced silencing complex (RISC), and the endonuclease Argonaute-2 (AGO2) cleaves the sense strand of siRNA. Afterward, the antisense strand of the activated RISC binds explicitly to the homologous region of the messenger RNA (mRNA) expressed by the exogenous gene. As a result, mRNA is cleaved by RISC at the binding site, causing the specific gene silence. The cleavage site is the two ends that complement the antisense strand. The cleaved fragmented mRNA is then degraded, thereby preventing the translation of mRNA and silencing gene expression in the host cell (Ichim et al., 2004; Rao et al., 2009) (Figure 1).

The target sequence recognition is the antisense strand of siRNA in RISC. In the process of RNAi, the first RISC inactive precursor with a molecular weight of 250 kD is produced (Tang, 2005). After that, it will form an active complex of 100 kD in the presence of ATP. The conversion from an inactive precursor to an active enzyme complex is similar to activating proteases. Still, the activation of the RISC enzyme complex does not require covalent bond cleavage. Instead, it involves unraveling the double strand of siRNA bound to the RISC. First, the ATP-dependent helicase unwraps the double strand of siRNA when ATP is present. And then, the target mRNA, which complements the antisense strand, replaces the sense strand of siRNA. Finally, the target mRNA sequence in the middle of the complementary region, about 12 bp away from the 3′end of the siRNA antisense strand, is cut by the activated RISC (Nykänen et al., 2001). siRNA can not only guide RISC to cut homologous single-stranded mRNA but be used as a primer binding to target RNA and synthesize more new dsRNA under the action of RNA-dependent RNA polymerase. The newly synthesized dsRNA is then cleaved by Dicer to produce many secondary siRNAs (Hutvágner and Zamore, 2002). After several synthesis-cutting cycles, RNAi’s role is continuously amplified, and finally, the target mRNA is completely degraded (Figure 1).

This natural defense mechanism implies that artificial induction of siRNA can effectively therapeutically silence pathological genes. In the case of externally introduced siRNA, siRNA can be directly loaded into RISC without pre-treatment with Dicer. Once loaded, the sense strand with the same nucleotide sequence as the mRNA is separated from the RISC complex and degraded, while the antisense strand is still attached to the RISC and guides the complex to the target mRNA (Li and Rana, 2012). The gene silencing mechanism of exogenous siRNA is similar to endogenous siRNA (Figure 1). The synthesis methods of siRNA include chemical and enzymatic synthesis (Ichim et al., 2004). Enzymatically generated siRNA can correspond to the sequence overlapping with the entire gene, which is more effective and convenient than chemically synthesized siRNA (Ichim et al., 2004).

## Hurdlers of Small Interfering RNA Delivery in Gliomas

Although siRNA therapy is a promising strategy for glioma treatment, there are some concerns regarding its applications in clinical therapy. siRNAs mainly be injected by systemic administration for glioma treatment. However, as soon as artificially prepared siRNA is administered to the blood, it will be degraded by the innate immune system and serum nucleases (Benitez et al., 2015). Additionally, exogenous siRNA may compete with endogenous RNA, resulting in oversaturation (Grimm et al., 2006). Exogenous siRNA may also cause “off-target effects” and, therefore, silence the expression of non-target functional genes; this is a pronounced side effect of siRNA (Jackson and Linsley, 2010; Marinho et al., 2018). siRNAs have a short half-life in the blood because they are easily stained by nucleases and filtered out by the kidney and liver (Choi et al., 2007). Therefore, free siRNAs cannot target tissues and tumors. Furthermore, siRNA is a kind of water-soluble macromolecule with a negative charge, and it is difficult to pass through the negatively charged cell membrane under normal circumstances (de Fougerolles et al., 2007). Even if it enters the cell through endocytosis, it will often enter the lysosome and be degraded (Dominska and Dykxhoorn, 2010). It is worth noting that the BBB is an additional limitation for the development of RNAi as a therapeutic strategy for brain tumors. BBB maintains proper ion levels, and brain nutrition and protects brain tissue from potential damage caused by chemical damage and nerve agents (Abbott et al., 2010). Endothelial cells, which compose most of the BBB’s capillaries lining, remain tightly sealed, mainly through tight junctions. These tight junctions are composed of barrier-forming proteins, such as claudins, occluders, junction adhesion molecules, and cytoplasmic accessory proteins (Bhowmik and Khan, 2015). These interactions only allow molecules with specific characteristics to cross the blood into the brain, excluding more than 98% of potential therapeutic drugs (Kumar et al., 2007).

Given the high degradation by enzymes in tissues and cells, along with the poor cellular uptake of naked siRNA, there is an urgent need for developing delivery vehicles to transport siRNA to achieve the optimal silencing effect of the target gene. These delivery systems must be engineered to 1) reduce the renal clearance to increase the retention time of siRNA in the circulatory system, 2) protect siRNA from degradation by serum nucleases, 3) ensure effective biodistribution, 4) effectively penetrate the BBB and enter the brain, 5) promote siRNA transport to target cells and promote uptake, and 6) promote trafficking to the cytoplasm and uptake into RISC.

## Delivery Strategies for Promising Small Interfering RNA Therapeutics in Gliomas

**FIGURE 2:**
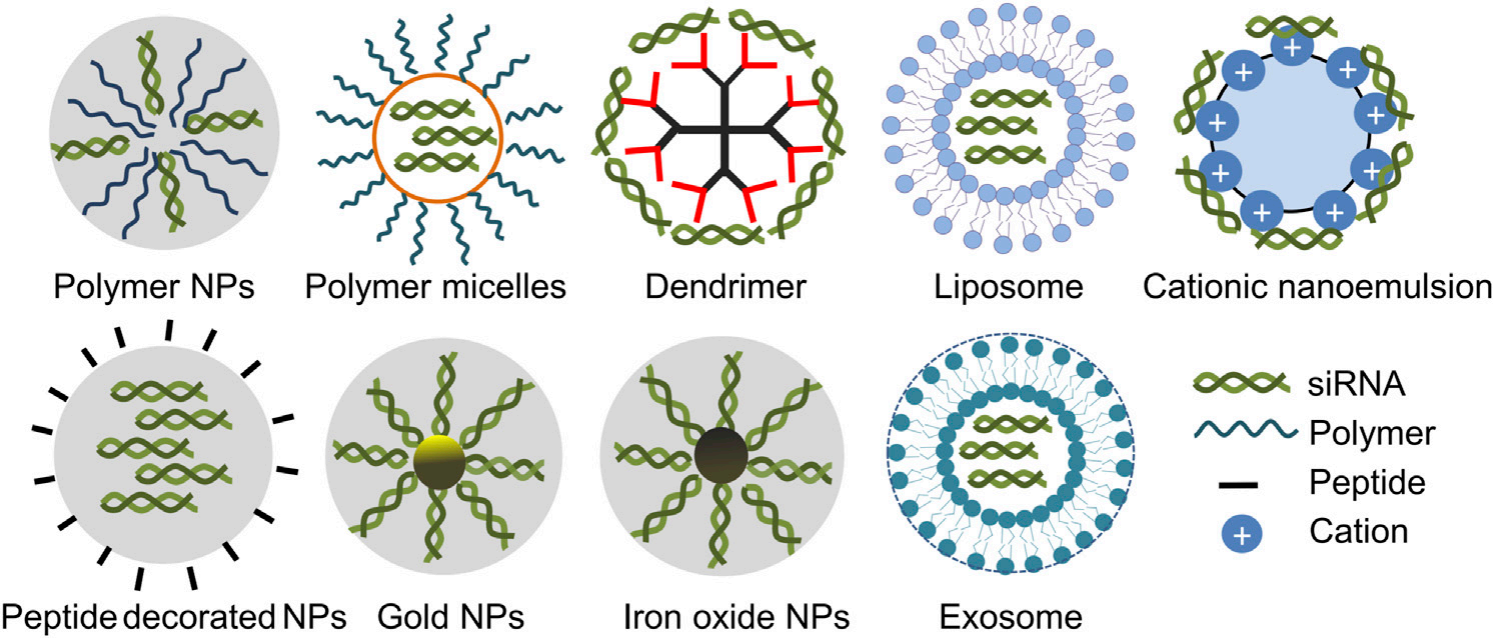
Simplified diagrams of some representative carriers applied for siRNA delivery in gliomas. siRNA carriers including polymer NPs (Karlsson et al., 2021), polymer micelles (Zhang et al., 2020a), dendrimer (Qiu et al., 2018), liposome (Hu et al., 2021), nanoemulsion (Teixeira et al., 2020), peptide decorated NPs (Zheng et al., 2019), gold NPs (Kumthekar et al., 2021), iron oxide NPs (Kievit et al., 2017) and exosome (Fu et al., 2021).

As discussed, employing siRNAs against oncogenes can be a promising procedure to combat different malignancies of gliomas. However, their clinical application is greatly restricted due to the obstacles to delivering siRNAs to glioma cells. Nowadays, the chemical modification of the siRNA backbone and the use of various delivery vehicles are considered the most promising techniques to deliver siRNA to the target cells (Gavrilov and Saltzman, 2012). Below, we describe a series of siRNA delivery technologies developed to improve glioma therapies’ stability, safety, and effectiveness and the advantages and application prospects of these delivery systems. The current research on siRNA delivery strategies is summarized in Table 1. The characteristics and advantages of these delivery strategies can be found in them. In addition, we also specify the use of cell lines and animals in Table 1 for specificity. The schematic diagram of some promising delivery strategies is shown in Figure 2.

**TABLE 1:** siRNA delivery strategies in gliomas.

Vectors	Targeted genes/proteins	Tumor model (glioma cells)	Characteristics	Advantages	References
Virus-derived vector	PD-1/PD-L1	Subcutaneous/brain GBM mouse model (C57BL/6N)	A non-replicating virus-derived vector	Robust antitumor immunity	Sugii et al. (2021)
Polymer-based NPs	STAT3	Orthotopic, syngeneic mouse model (Tu2449 gliomas)	PEI-based lipopolyplex	Enhanced transfection efficiencies, decreased cytotoxicity and high colloidal stability, biocompatibility,	Linder et al. (2019)
	Gli1	Subcutaneous tumor-bearing mice	Copolymer self-assembly	High transfection performance (98%)	Zhou et al. (2018)
	EZH2	Glioma (U251) and glioma stem cell lines (NSC11, GBMJ1)	A new type of nanomaterial ECO	Radio-sensitzation, selective	Lee et al. (2020)
	Bcl-2	Patient-derived cancer cell lines and in murine GBM	Adding photocrosslinked polymers to cationic polymer PBAE	High serum stability, cell uptake and tissue-mediated delivery to extrahepatic tissues, effective endosomal escape	Karlsson et al. (2021)
	Robo1, YAP1, NKCC1, EGFR, survivin	Primary human GBM cells versus primary human neural progenitor cells	Boreducible linear BR6-S4 polymer	Biodegradable polymer and cancer-selective	Kozielski et al. (2019)
	CD146	Subcutaneous and intracranial gliomas	Chitosan oligosaccharide lactate NPs conjugated with folic acid-polyethylene glycol.	Accumulation in subcutaneous and intracranial gliomas, suppression of intracranial tumor growth	Fukui et al. (2020)
	VEGF	U87MG cells	cRGD modified PEG conjugated to the HA2 modified chitosan *via* a hydrazone linkage.	Accurately target glioma cells, assists the escape from endosome into cytosol	Zhang et al. (2020a)
Dendrimer	YKL40, Arg1, Id1, MMP14, cMyc, CX3CR1	Primary microglia	Amphiphilic dendrimer	Low toxicity, efficiently delivery	Ellert-Miklaszewska et al. (2019)
	p42	Glioma (C6), and GBM (U87)	Cationic dendrimers based on 2,2-bis(methylol) propionic acid	Highly efficient at endo-lysosomal escape	Stenström et al. (2018)
	Bcl-2, VEGF	U87	Dendrimer-entrapped gold NPs conjugated with β-cyclodextrin	Efficiently delivery and good cytocompatibility	Qiu et al. (2018)
	CLTC, CAV1, and PAK1	C6, U87, GL261 and T98G	β-CD-based molecular multivalent amphiphile AMC6	High transfection efficiency	Manzanares et al. (2020)
Lipid-based NPs	c-Myc	Orthotopic glioma	A lipoplex encapsulation by liposome modified with peptide	Penetrate nasal mucosa, selectively internalized, increased distribution, avoid premature release	Hu et al. (2021)
	CD163	Nude mice bearing intracranial gliomas (U87, LN229 cell)	pH-sensitive PEG-ligand-lipid	Bypassed the BBB, cope with different environments,	Liu et al. (2020a)
	CD73	C6 glioma cells and primary astrocytes	Cationic nanoemulsions	High delivery efficiency and specificity	(Teixeira et al., 2020) (Azambuja et al., 2020b)
Peptide decorated NPs	PLK1, VEGF2	Orthotopic human GBM tumor-bearing nude mice (U87MGLuc)	Stabilized by triple interactions, angiopep-2 peptide modified	Superb BBB penetration and potent tumor accumulation	Zheng et al. (2019)
	PLK2	Mouse xenograft and patient-derived xenograft models (H441-luc cells)	Tyrosine modified PEI	Good physical, chemical properties and very high biocompatibility	Karimov et al. (2021)
	PLK1	GBM carrying mice (U-87 MG)	Angiopep-2 peptide-decorated chimaeric polymersomes	Prolonged the circulation time of siRNA and enhanced its accumulation in cancer	Shi et al. (2018)
	PLK1	Xenografts (U87MG)	Angiopep-2 functionalized intracellular-environment-responsive siRNA nanocapsule	Long circulation in plasma, efficient BBB penetration capability, GBM accumulation and retention, responsive intracellular siRNA release	Zou et al. (2020)
	HDGF	Nude mice bearing human GBM (U251)	Peptide-modified pH-sensitive self-assembled hybrid NPs	Against degradation by serum nucleases and substantially improved the stability	Zhou et al. (2019)
	STAT3	Patient-derived gliospheres (BT-13)	Chemically modified pluronic F108 as an amphiphilic polymer, conjugated the MDGI receptor targeting COOP peptide	Enhanced transfection efficiencies	Kadekar and Nawale (2021)
	EGFR	Xenograft tumor-bearing mice (U87)	PVBLG-8 with a rigid, linear structure, and PLG with a flexible chain is incorporated as a stabilizer	Promote tumor penetration, selective cancer cell internalization and effective endolysosome escape	Liu et al. (2018)
	PLK1	Orthotopic human GBM tumor-bearing nude mice (U87MG-luc)	A three-layer core-shell structure uses Angiopep-2 peptide-modified, immune-free red blood cell membrane and charge conversion components	Good biocompatibility, prolonged blood circulation, high BBB transcytosis, effective tumor accumulation, and specific uptake by tumor cells in the brain	Liu et al. (2020b)
Inorganic NPs	Bcl-2	Xenografted tumors (U87MG cells)	PEI-entrapped gold NPs modified with an RGD peptide *via* a PEG spacer	Excellent biocompatibility, highly efficient transfection efficiency and specific targeting properties	Kong et al. (2017)
	Bcl2Like12	Subcutaneous and orthotopic xenografts (HROC24)	SNAs consist of gold NP cores covalently conjugated with radially oriented and densely packed siRNA oligonucleotides	Robust penetration of SNAs into brain and tumor through transcytosis	Kumthekar et al. (2021)
	MRP1	Tumor-bearing mice (U-87)	Porous silicon NPs with PEI capping	Biocompatible, high-capacity loading of siRNA, and optimized release profile	Tong et al. (2018)
	VEGFA	GBM orthotopic model (U87MG)	RGD peptide-decorated BSA was employed as the stabilizer and scaffold to fabricate Mn(iii)- and Mn(iv)-integrated NPs	Good stability, excellent biocompatibility, increased tumor uptake, improved tumor accumulation and enhanced therapeutic effects with the modulation of the TME	Xu et al. (2020)
	REST	U87 and U251 GBM cells	PEI-coated Fe₃O₄ NPs	Effective siRNA delivery	Wang and Degirmenci, (2018)
	Ape1	Genetic mouse model of GBM	Superparamagnetic iron oxide core coated with a copolymer of chitosan, PEG, and PEI, functionalized with the tumor-targeting peptide	Stably bind and protect nucleic acids for specific delivery into brain tumor cells	Kievit et al. (2017)
Exosomes	F3-T3	Glioma-bearing mice	Mesenchymal stem cell-derived exosomes	Avoiding normal tissue toxicity	Parker Kerrigan et al. (2020)
	EGFR/TNC	Nude mouse orthotopic tumor model (U87MG-Luc)	Use the liver as a tissue chassis to direct the self-assembly of exogenous siRNAs into secretory exosomes	Efficiently pass through the BBB	Fu et al. (2021)
siRNA-conjugated system	EGFR	Nude mice bearing a tumor xenograft (U87MG)	Conjugate comprising methoxy-modified siRNA and cRGD peptides	High targeting ability, substantial anti-tumor effects and low toxicity	He et al. (2017)
Aptamer	Survivin	U87 cells	Modifying one side of the DNA nanostructure with aptamer as1411	Selectively recognize the nucleolin in the cytomembrane of tumor cells.	Zhou et al. (2021)

Notes: NPs, nanoparticles; RGD, arginine-glycine-aspartic (arg-gly-asp); BBB, blood-brain barrier; GBM, glioblastoma; BSA, bovine serum albumin; SNAs, spherical nucleic acids.

### Viral Vectors

Various strategies have been applied to develop effective siRNA delivery materials in gliomas to overcome obstacles responsible for transfection efficiency, cell toxicity, and cell specificity. Viral vectors are popular for siRNA delivery due to their practical ability to infect host cells. Currently, commonly used viral vectors include retroviruses, lentiviruses, adenoviruses, adeno-associated viruses, and herpes simplex viruses. For example, programmed cell death-1 (PD-1) is an immune checkpoint molecule expressed on the cell surface of activated lymphocytes. When PD-1 binds with its ligand, programmed cell death-ligand 1 (PD-L1), an inhibitory signal is transduced, and immune cells become inactivated or exhausted. And PD-L1 is abundantly expressed in GBM (Xue et al., 2017). Researchers used a non-replicating virus-derived vector, a hemagglutinating Japan-envelope (HVJ-E) virus, to inhibit PD-L1 expression by delivering siRNA. And it provokes robust antitumoral immunity by activating natural killer cells and cytotoxic T lymphocytes and suppressing regulatory T lymphocytes. Thus, HVJ-E may be a new treatment option for GBM patients (Sugii et al., 2021). However, viral vectors have many defects which limit their clinical applications, such as high cytotoxicity and immunogenicity, ease of causing inflammation, high cost, limited size, and quantity of loaded DNA. Therefore, viral vectors need further study for clinical application.

### Polymeric Nanoparticles

**FIGURE 3:**
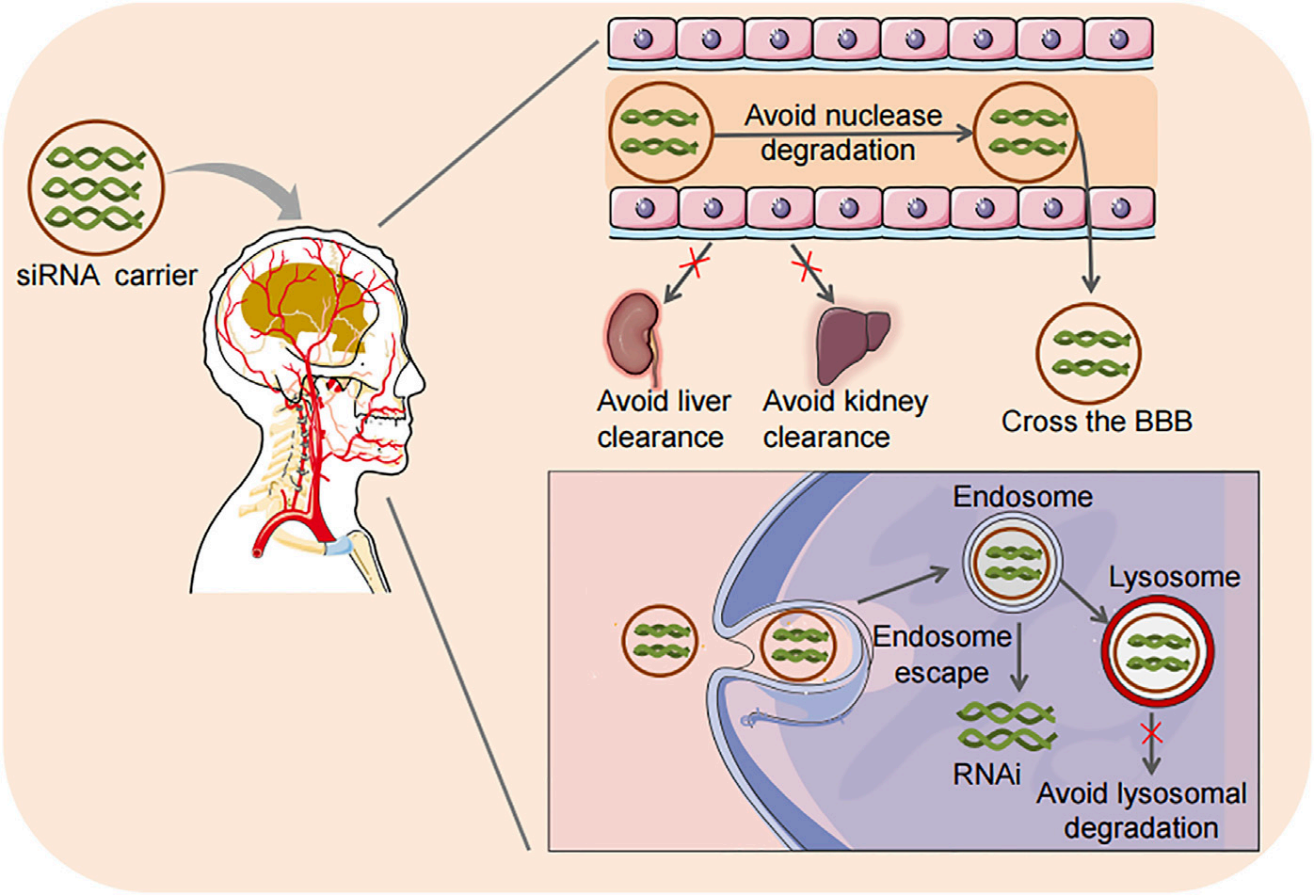
Advantages of siRNA delivery strategies. Loading siRNA into carriers has the advantages of preventing nuclease degradation, reducing the clearance by the kidney and liver, effectively crossing the BBB, facilitating endosome-lysosome escape, and avoiding lysosome degradation in cells. RNAi, RNA interference.

Given the deficiencies of viral vectors, non-viral delivery systems are getting more attention due to their low toxicity, potential for targeted delivery, long-term stability, high loading, controllable chemical structure, low immunogenicity, easy mass production, and relatively low production costs. Therefore, non-viral vectors for siRNA delivery have been extensively studied in gliomas in recent years. Nano-materials are considered the most promising non-viral carriers for siRNA delivery. A variety of nanoparticles (NPs), including polymeric NPs, lipid-based NPs, peptide NPs, inorganic NPs, etc. have been reported for siRNA delivery in glioma therapy. Delivery vehicles mainly deliver siRNA to endo/lysosomal vesicles. Effective drugs must then escape from endo/lysosomal vesicles into the cytoplasm and translocate into the nuclei (Rajendran et al., 2010). The intracellular transport of siRNA delivered by nanocarriers usually starts in the early endosomes, and then the contents are transferred to the late endosomes. The endosomal compartment of the cell is strongly acidic, while the cytoplasm or intracellular space is approximately neutral. Then the endosome is relocated to the lysosome and further acidified, promoting siRNA degradation (Dominska and Dykxhoorn, 2010). More importantly, the nanocarrier delivery system can be designed to facilitate endosome-lysosome escape. The advantages of nanocarrier delivery systems are shown in Figure 3.

Polymer-based NPs are a well-studied system for siRNA delivery. The ease of synthesis and flexibility feature makes them one of the main non-viral gene delivery vectors. Polymer-mediated siRNA delivery to cancer cells has several advantages. For instance, the electrostatic complex between the positively charged polymer and the phosphate group of the negatively charged siRNA backbone provokes siRNA condensation, thereby avoiding nuclease degradation. Furthermore, it can be made into soluble derivatives with good biocompatibility after chemical modification. And it can be conjugated with inorganic materials. In this way, various obstacles related to effective siRNA delivery can be resolved. In addition, cationic polymers with secondary and tertiary amines have shown significant buffering capacity, which helps the endosomal escape of siRNAs (Karlsson et al., 2021).

Polyethylenimine (PEI) is a branched cationic polymer that has shown remarkable effects in transfecting DNA into cells with superior tolerance, but they show poor siRNA complexing (Karimov et al., 2021). Therefore, it is necessary to find new ways to improve the delivery efficiency of PEI. For example, the oncogenic transcription factor signal transducer and activator of transcription 3 (STAT3) is a key molecule that is often highly expressed and overactivated in GBM and is associated with the most aggressive and therapeutically resistant mesenchymal subtype (Yu et al., 2014). Benedikt (Linder et al., 2019) and colleagues formulated siRNAs in nanoscale lipopolyplexes based on PEI and the phospholipid 1,2-dipalmitoyl-sn-glycero-3-phosphocholine, which represents a promising new approach to target STAT3 in glioma. Results showed decreased tumor growth and significantly prolonged survival in Tu2449 glioma-bearing mice. However, PEI is not biodegradable and has severe cytotoxicity, limiting its use (Wang et al., 2019). Although the toxicity of PEI can be reduced by introducing hydrophobic characters, it could affect the polyplexes formation with siRNA (Charbe et al., 2020). Thus it is needed to find new polymers for siRNA delivery.

Glioma-related oncogene homolog 1 (Gli1) was originally named in glioma research, which can affect glioma cell apoptosis and proliferation *via* regulated cyclin D1 and Bcl-2 (Du et al., 2013). Therefore, Gli1 is an ideal candidate target for gliomas gene therapy. Zhou (Zhou et al., 2018) and colleagues developed a novel gene delivery system with a self-assembly method using a1, 2-dioleoyl-3-trimethylammonium-propane and methoxy poly (ethylene glycol)-poly (lactide) copolymer (DMP), which showed good performance in delivering siRNA to glioma cells *in vitro* with high transfection performance, and the treatment with the DMP-Gli1siRNA complex for subcutaneous tumor-bearing mice significantly inhibited tumor growth by promoting apoptosis and reducing proliferation. Similarly, EZH2 is another ideal candidate for tumor gene therapy, which is overexpressed in many tumors (Lu et al., 2010). Reducing the expression of EZH2 in tumor cells could inhibit proliferation, migration, invasion, angiogenesis, and induce apoptosis (Lu et al., 2010). Wang and colleagues (Wang et al., 2019) developed a biodegradable NPs delivery system for EZH2 siRNA with a self-assembly method by methoxy polyethylene glycol-polycaprolactone and DOTAP (DMC). Treatment of tumor-bearing mice with DMC-EZH2 siRNA complex significantly inhibited tumor growth *in vivo*. In line with this, Lee (Lee et al., 2020) and colleagues used a new nanomaterial ECO to deliver siRNA targeting DNA damage response proteins ataxia telangiectasia mutated and DNA-dependent protein kinase (DNApk-cs) for the radiosensitization of GBM *in vitro* and *in vivo*. ECO NPs efficiently delivered siRNA and silenced target protein expression in glioma (U251) and glioma stem cell lines (NSC11, GBMJ1). Importantly, ECO NPs are selective that have little effect on normal astrocytes.

Poly (β-amino ester)s (PBAE)s are one class of biodegradable cationic polymers. Adding photocrosslinked polymers to the PBAE structure produces particles with neutral surface charges that can be used in nucleic acid-based therapies. This polymer nanocarrier contains ester bonds for hydrolytic degradation and disulfide bonds for environmentally triggered siRNA release in the cytosol. Such NPs can effectively achieve siRNA-mediated knockdown in gliomas and exhibit high serum stability, cell uptake, and tissue-mediated delivery to extrahepatic tissues. Moreover, the presence of both secondary and tertiary amines of these NPs leads to efficient buffering at low pH, leading to endosomal disruption, which achieves effective endosomal escape (Karlsson et al., 2021).

Furthermore, polymer NPs can also be used to target multiple genes simultaneously. Kozielski and colleagues *via* polymerization of monomer bis(2-hydroxyethyl) disulfide (BR6) with monomer 4-amino-1-butanol (S4), followed by polymer end-capping with 2-((3-aminopropyl)amino)ethanol (E6) synthesized NPs, which are synthetic, bioreducible, and biodegradable polymer that can package and deliver hundreds of siRNA molecules into a single NP, facilitating the promotion of multiple GBM targets. And the siRNA delivery of these polymer NPs is cancer-selective, thereby avoiding potential side effects on healthy cells (Kozielski et al., 2019).

Chitosan is a natural polymer with biocompatibility and low immunogenicity. However, it is necessary to modify the structure action to overcome the particle degradation and low delivery efficiency of naked chitosan. CD146 is mainly expressed in dividing glioma stem cells and regulates cell cycle progression. Naoki and colleagues designed chitosan-oligosaccharide lactic acid NPs conjugated with folic acid-polyethylene glycol to deliver CD146 siRNA. They found that these NPs targeting CD146 can inhibit tumor growth in the mouse glioma model (Fukui et al., 2020). Similarly, vascular endothelial growth factor (VEGF) is an essential signaling molecule during angiogenesis (Guan et al., 2005). RNAi-mediated silencing of VEGF expression can selectively inhibit target gene expression and result in decreased blood vessel density and delayed tumor growth (Guan et al., 2005). Novel pH-responsive and virus mimetic shell-sheddable chitosan (CS) micelles (CMs) have been reported for VEGF siRNA delivery. The cRGD modified PEG was conjugated to the HA2 modified chitosan *via* a hydrazone linkage (cRGD-PEG-Hz-CS-HA2), then loaded the siVEGF payload into the core of cRGD/HA2/Hz-CMs through electrostatic interaction and hydrophobic interaction. siVEGF was released by the pH-responsive cleavage of the hydrazone bond in the acidic environment of the endosome. And the exposed HA2 acted as a pH-sensitive membrane destroying peptide, which helps the carrier escape from the endosome into the cytoplasm, effectively silencing VEGF gene expression in U87MG cells (Zhang et al., 2020a).

### Dendrimers

Dendrimers are symmetric macromolecules characterized by their 3-D spherical structure, nano-scale size, and monodisperse properties. They have flexible structures that encapsulate anti-cancer drugs either in the core or surface. Studies have shown that dendrimers can be developed as carriers for siRNA delivery. Microglia, the brain-resident macrophages, which contribute to the progression of brain tumors, including GBM, are considered a potentially promising therapeutic target (Hambardzumyan et al., 2016; Poon et al., 2017). Researchers employed an amphiphilic dendrimer nanocarrier with low toxicity effectively to deliver siRNA to primary microglia and decreased target gene and protein expression, leading to transcriptomic changes without affecting basal microglial functions (Ellert-Miklaszewska et al., 2019).

Cationic dendrimers based on 2,2-bis(methylol)propionic acid have also been reported as nonviral carriers for siRNA transfection. These dendrimers are highly efficient at endo-lysosomal escape (Stenström et al., 2018). However, how this kind of dendrimer achieves endosomal escape has not been studied. Poly (amidoamine) (PAMAM) dendrimers are a class of monodispersed macromolecules with branched interior and well-fixed molecular conformation, and controlled surface functionalities (Shakhbazau et al., 2010). They are a type of nano-molecules ranging in size (between 1 and 100 nm) that have the advantage of delivering siRNA and crossing the BBB with low cytotoxicity (Fana et al., 2020). Additionally, cyclodextrin modification has also been used to improve the delivery efficiency of siRNA. Qiu and colleagues (Qiu et al., 2018) utilized amine-terminated generation 5 PAMAM dendrimers partially grafted with β-cyclodextrin (β-CD) as a nanoreactor to entrap gold NPs (Au DENPs). They obtained β -CD modified Au DENPs (Au DENPs-β-CD) and combined them with two different types of therapeutic siRNA: B-cell lymphoma/leukemia-2 (Bcl-2) siRNA and VEGF siRNA. Research data reveals that the formed Au DENPs-β-CD carrier enables efficient delivery and good cytocompatibility and enables enhanced gene silencing to inhibit the expression of Bcl-2 and VEGF proteins (Qiu et al., 2018). In line with this, Manzanares and colleagues used a β-CD-based molecular multivalent amphiphile AMC6 as a carrier to deliver siRNA. In fact, most NPs enter cells mainly through three endocytosis pathways: macropinocytosis (Mercer and Helenius, 2012), clathrin-mediated endocytosis (CME) (McMahon and Boucrot, 2011) and caveolin-mediated endocytosis (CVME) (Pelkmans et al., 2004). Researchers then used siRNA to knock down the key protein involved in macropinocytosis (PAK1), resulting in a 35% reduction in cellular intake. However, the knockout of the key proteins involved in CME and CVME did not reduce intake. These results showed that the nanocomplexes composed of siRNA and AMC6 could preferentially deliver siRNA to GBM cells efficiently through macropinocytosis (Manzanares et al., 2020).

### Lipid-Based Nanoparticles

Lipid-based NPs (LNPs) systems are usually less than 100 nm in diameter. Since the cell membrane mainly comprises lipids and phospholipids, LNP interacts with the cell membrane (Chen et al., 2018). LNPs are non-toxic, biocompatible, non-immunogenic, and soluble agents, becoming a favorable platform for siRNA delivery (Itani and Al Faraj, 2019). LNPs for drug delivery include liposomes, micelles, and nanoemulsions (NEs). Liposomes can entrap hydrophilic molecules in their aqueous core, whereas the hydrophobic molecules get trapped inside the lipid layer. LNPs can protect the encapsulated siRNA from nuclease degradation and renal clearance and promote siRNA’s cellular uptake and endosomal escape (Xu and Szoka, 1996).

Nowadays, for siRNA-based glioma treatment *in vivo*, various routes of administration have been explored, including intranasal administration. Lipid-based NPs have been used for intranasal administration. For example, to promote intranasal delivery of c-Myc siRNA for GBM treatment, Hu and colleagues (Hu et al., 2021) constructed a lipoplex based on pre-compression of c-Myc-targeting siRNA by octaarginine and subsequent encapsulation by liposome modified with a selected peptide derived from penetratin. This lipid complex exhibits a stable core-shell structure, avoiding being trapped by lysosomes and releasing siRNA in the cytoplasm. Furthermore, due to significantly enhanced permeability in tumor spheroids and nasal mucosa, the lipoplex was competent to deliver more siRNA to orthotopic glioma after intranasal administration. Therefore, it prolonged the survival time of glioma-bearing mice by inducing apoptosis.

NEs are particularly advantageous for cancer therapy applications due to their high delivery efficiency and specificity (Fraga et al., 2015). Ecto-5′-nucleotidase (CD73) is a glycosylphosphatidylinositol-anchored nucleotidase that catalyzes the hydrolysis of extracellular AMP to adenosine (Allard et al., 2019). CD73 siRNA can inhibit glioma growth *in vivo* and potentiate TMZ cytotoxic effect on glioma cells (Azambuja et al., 2019). It is reported that the cationic NE loaded with CD73 siRNA can serve as an effective treatment strategy for gliomas. Studies have shown that intravenous delivery of these NEs effectively suppressed the activity of CD73 and AMPase, accompanied by reducing the viability of glioma cells (Teixeira et al., 2020). Furthermore, other researchers used NE loaded with CD73 siRNA to treat GBM-bearing rats nasally, which was found to potentially regulate the immune microenvironment of GBM and delay tumor growth by inducing apoptosis (Azambuja et al., 2020b). Subsequently, NE loaded with CD73 siRNA and TMZ were combined for nasal administration. Unexpectedly, no synergistic or additive *in vivo* was promoted (Azambuja et al., 2020a).

Smart nanocarriers such as those responsive to pH can be developed in siRNA delivery. Lipids complexed with polymers, mostly PEG, have been reported to prevent the immune system from recognizing liposomes and reduce liver clearance (Liu et al., 2020a). CD163 was reported to regulate cell proliferation and self-renewal of gliomas *via* casein kinase 2 (Chen et al., 2019). For co-delivering of shCD163 and doxorubicin (DOX) in gliomas, Liu and colleagues synthesized a pH-sensitive PEG-ligand-lipid, DSPEcRGD-Hz-mPEG9. This nanodrug-loading platform is a lipid-polymer hybrid NPs that provides the PEG shell during its circulation in the blood. In the acidic glioma microenvironment, the PEG shell of the cRGD lipid would shed, exposing the cRGD lipid and facilitating the recognition of the cRGD lipid by cells and the drug release. Moreover, *in vivo* studies proved that it can inhibit the growth and stemness of glioma cells and prevent the recurrence of gliomas after resection (Liu et al., 2020a).

Recently, Liu et al. (2020b) developed another charge conversional biomimetic nanoplatform with a three-layer core-shell structure to programmatically overcome the persistent barriers to delivering siRNA in GBM. Angiopep-2-decorated red blood cell membrane serves as the outside shell; citraconic anhydride grafted poly-Llysine is used as the middle layer with a negative charge for pH-triggered charge conversion, and the inner core is PEI complexes formed *via* electrostatic interaction for siRNA condensing and protection. This nanoplatform enables good biocompatibility, prolonged blood circulation, high BBB transcytosis, effective tumor accumulation, and specific uptake by tumor cells in the brain. In addition, red blood cell membrane destruction and effective siRNA release can be further triggered by the conversion of negative charges in tumor cells/lysosomes (pH 5.0–6.5) to positive charges, resulting in efficient target gene silencing and strong resistance. A higher therapeutic effect and survival have been shown in orthotopic U87MG-luc human GBM tumor-bearing nude mice.

### Peptide Nanoparticles

A key barrier to design more efficient NPs for crossing the BBB while protecting its cargo from release and degradation is that no explicit knowledge of the main molecular factors involved in efficient nanocarrier crossing of BBB is available. The general approach to reach this goal is to decorate the nanocarriers by targeting ligands to mainly endothelial cells as critical components of BBB. Ligands like cell-penetrating peptides have been used. For example, Gregory and colleagues engineered another vector synthetic protein NP for STAT3 siRNA delivery. These NPs are decorated with cell penetration peptide iRGD, which can effectively overcome the limitations of the BBB (Gregory et al., 2020). In addition, using peptides, polymers and siRNA co-complexed to form nanocarriers also shows excellent tumor penetration (Liu et al., 2018).

Zheng (Zheng et al., 2019) and colleagues constructed a polymeric siRNA nanomedicine stabilized by triple interactions (electrostatic, hydrogen bond, and hydrophobic). The developed nanomedicine shows effective at-site siRNA release caused by continuous instability triggered by tumor reactive oxygen species (ROS). In addition, the angiopep-2 peptide is used to enhance the utility of this nanomedicine for GBM treatment, which exhibits superb BBB penetration and effective tumor accumulation. Angiogenesis is recognized as a principal hallmark of various cancers, including GBM. Co-targeting PLK1 and VEGF receptor-2 by this polymeric siRNA nanomedicine can effectively suppress tumor growth and improve the survival time of nude mice bearing orthotopic GBM (Zheng et al., 2019).

Serine/threonine-protein kinase polo-like kinase 2 (PLK2) is a key regulator that participates in centriole duplication (Warnke et al., 2004) and G1/S phase transition (Chang et al., 2010). Alafate et al. (2020) reported that loss of PLK2 can enhance aggressive biological behavior of GBM through activation of Notch signaling, indicating that PLK2 may be a potential therapeutic target for GBM. Researchers used tyrosine to modify the small linear PEI, which showed good physical and chemical properties and high biocompatibility. The tyrosine-modified PEI and PLK2 siRNA complexes have shown anti-tumor effects in mouse xenografts and patient-derived xenografts models (Karimov et al., 2021).

Similarly, Angiopep-2 peptide modified chimeric polymer (ANG-CP) has been reported as a non-toxic and non-viral vector targeting the brain to promote RNAi treatment of human GBM *in vivo* (Shi et al., 2018). ANG-CP shows excellent packaging and protection against PLK1 siRNA in its lumen while rapidly releasing the payload in the cytoplasmic reduction environment. *In vitro* experiments showed that ANG-CP significantly silenced PLK1 mRNA and corresponding oncoprotein in U-87 MG cells and greatly prolonged the circulation time of PLK1 siRNA, and enhanced its accumulation in GBM. *In vivo* experiments proved that ANG-CP carrying PLK1 siRNA significantly increased the survival time of mice with GBM. Furthermore, Zou and colleagues (Zou et al., 2020) successfully designed another Angiopep-2 (Ang) functionalized intracellular-environment-responsive siRNA nanocapsule to target PLK1. Due to the unique design of small size (25 nm) with a polymeric shell for siRNA protection, this nanocapsule displayed long circulation in plasma, efficient BBB penetration capability, GBM accumulation and retention, and responsive BBB penetration capability intracellular siRNA release. Ang peptide was linked to nanoparticles *via* amidation chemical reaction, enabling BBB crossing and GBM targeting. Furthermore, the disulfide bonds have a good response to the redox environment of the cell, which is mainly because the concentration of reduced glutathione in the cytoplasm and cell endosomes is much higher than it in the plasma (Wu et al., 2004). Therefore, the disulfide bonds can exist stably in the oxidized extracellular environment, but are unstable in the reductive intracellular environment. The disulfide bonds help achieve the goal of stable delivery and efficient release of siRNA (Bauhuber et al., 2009). Moreover, this nanocapsule showed outstanding growth inhibition of orthotopic U87MG xenografts.

Hepatoma-derived growth factor (HDGF) is an acidic heparin-binding protein with mitogenic and angiogenic functions (Tang et al., 2016), whose expression level in gliomas is positively correlated with the degree of malignancy. HDGF is considered a potential therapeutic target for gliomas (Zhou et al., 2019). Zhou et al. (2019) developed a nanocomposite containing a PLGA core and a PEG shell, and modified its surface with the peptide H7K(R2)2 composed of the polyhistidine peptide (H7), the two-branched-chain arginine peptide (R2)2 and the lysine (K) linker between the two amino acid sequences, to deliver siHDGF. Following systemic administration, it can effectively deliver siHDGF into the brain and malignant glioma cells and against degradation by serum nucleases and substantially improve its stability, resulting in reduced tumor volumes and prolonged survival times in nude mice bearing U251 human GBM. Similarly, Kadekar and Nawale (2021) developed a siRNA delivery platform using chemically modified pluronic F108 as an amphiphilic polymer with a releasable bioactive disulfide functionality. They then conjugated the MDGI receptor targeting COOP peptide on the particle surface. Results suggested that transfection experiments with this design showed better silencing efficiency than the peptide’s absence.

In addition, peptides can also be used for siRNA delivery by complexation with siRNA. Liu (Liu et al., 2018) and colleagues reported a metastable, cancer-targeting siRNA delivery system based on two functional polymers PVBLG-8 (a cationic spiral cell penetrating polypeptide) and poly (l-glutamic acid) (PLG) (an anionic random-coiled polypeptide). PVBLG-8 with a rigid, linear structure shows weak siRNA condensing ability. When PLG and siRNA were co-complexed with PVBLG-8, PLG acted as a physical crosslinker to facilitate and stabilize the formation of NPs. The obtained positively charged NPs were sequentially coated with an additional amount of PLG, and the surface charge was reversed from positive to negative to obtain metastable NPs. These NPs have serum stability and can enhance tumor accumulation. After acidification in the extracellular microenvironment and endosome of the tumor, the partial protonation of PLG on the surface of NPs will cause the PLG coating to separate from the NPs, and the cationic, membrane-penetrating PVBLG-8 will be exposed. The surface charge will turn negative to positive, promoting tumor penetration, selective cancer cell internalization and effective endolysosome escape. Epidermal growth factor receptor (EGFR) is overexpressed in gliomas and associated with rapid proliferation and invasion (Guo et al., 2017). When EGFR siRNA is encapsulated in these NPs, it shows excellent tumor penetration, cell uptake level, EGFR silencing efficiency and tumor growth inhibition efficacy in U-87 MG GBM tumor spheroids *in vitro* and in xenograft tumor-bearing mice *in vivo*.

### Inorganic Nanoparticles

Inorganic NPs such as gold, iron oxide, silicon, Mn etc., have many promising biological and biomedical applications and are currently under intensive investigation to exploit the properties that make them good candidates for clinical applications in the future. Gold NPs are the most widely studied materials for siRNA delivery due to their inertness, nontoxicity, and biocompatibility properties. Furthermore, larger gold NPs (50 nm spheres and 40 nm stars) showed higher potential for siRNA delivery since larger particles deliver a greater amount of siRNA per particle (Yue et al., 2017). However, the underlying cause for these differences and the consequences on the function of siRNA delivered by NPs require further study.

Modifying inorganic NPs with cell penetrating peptides can achieve better delivery efficiency. For example, Kong and colleagues (Kong et al., 2017) reported using PEI-entrapped gold NPs modified with an RGD peptide *via* a PEG spacer as a vector for Bcl-2 siRNA delivery to GBM cells. The designed RGD-targeted gold NP is a novel vector system with excellent biocompatibility and efficient transfection efficiency. Own to the presence of RGD peptide, this vector system enables specific targeting properties.

Furthermore, Kumthekar et al. (2021) developed brain-penetrant RNA interference-based spherical nucleic acids (SNAs), consisting of gold NP cores covalently conjugated with radially oriented and densely packed siRNA oligonucleotides. GBM oncogene Bcl2Like12 (Bcl2L12) is a proline-rich protein characterized by a C-terminal 14 amino acid sequence with significant homology to the Bcl-2 Homology 2 domain found in several members of the Bcl-2 family (Scorilas et al., 2001). To determine the safety, pharmacokinetics, intratumoral accumulation, and gene-suppressive activity of systemically administered SNAs carrying siRNA specific for Bcl2L12, Kumthekar and colleagues conducted toxicology and toxicokinetic studies in nonhuman primates and a single-arm, open-label phase 0 first-in-human trial (NCT03020017). As a result, gold enrichment was observed in tumor-associated endothelium, macrophages, and tumor cells, and the expression of tumor-associated Bcl2L12 protein was reduced (Kumthekar et al., 2021). However, gold NPs are not easily degraded *in vivo*, limiting their clinical application.

Silicon NP is another promising inorganic nanocarrier for siRNA delivery, and compared with gold NP; it has better biological safety. Tong and colleagues established biocompatible porous silicon NPs (pSiNPs) with PEI capping that enable high-capacity loading of siRNA and optimized release profile (70% released between 24 and 48 h). It is well known that chemoresistance results from the expression of membrane-bound efflux transporters, such as the multidrug resistance protein (MRP) superfamily (Dallas et al., 2006). Multidrug resistance-related protein 1 (MRP1) is a subtype of MRP, whose overexpression has been proved to raise chemotherapy resistance of TMZ in brain tumors (Tivnan et al., 2015). And MRP1 silencing in GBM using MRP1-siRNA loaded pSiNPs was demonstrated in the mice model (Tong et al., 2018).

Mn(iv)-based NPs can effectively improve tumor oxygenation (hypoxia) and reduce endogenous hydrogen peroxide and acidity in the tumor area. However, Mn(iv)-based NPs are unstable in the acidic microenvironment, while the usual pH range of the tumor microenvironment (TME) is 6.5–7.0, which may limit their further application (Neri and Supuran, 2011). Researchers thus introduced Mn(iii) into the nanoplatform, reduced to Mn(ii) in a weakly acidic environment. They used sulfo-SMCC to link the RGD and bovine serum albumin through the typical procedure, which serves as a stabilizer and scaffold. Mn(iii) and Mn(iv) integrated nanocomposites were prepared with good stability and excellent biocompatibility. These NPs show superior advantages of siRNA delivery in gliomas, including increasing tumor uptake, improving tumor accumulation, and enhancing the therapeutic effect by adjusting TME (Xu et al., 2020).

A recent study reported that PEI-coated Fe_3_O_4_ NPs, characterized as magnetic NPs with a positive charge, enable effective siRNA delivery to target GBM cells (Wang and Degirmenci, 2018). The repair effect of the base excision repair (BER) pathway makes the radiotherapy effect of many tumors poor. Apurinic endonuclease 1 (Ape1) is critical to the function of BER, which cuts abasic damage for repair, so Ape1 is an ideal target for enhancing the effect of radiotherapy (Fung and Demple, 2011). Furthermore, Ape1 expression in GBM cell lines is predictive of radiosensitivity (Naidu et al., 2010). Forrest and colleagues (Kievit et al., 2017) developed NPs comprising a superparamagnetic iron oxide core coated with a copolymer of chitosan, PEG, and PEI to deliver anti-Ape1 siRNA to GBM cells and tumors. In addition, to improve the uptake by tumor cell surface, the NP was functionalized with the tumor-targeting peptide, chlorotoxin. Results showed that NP-mediated knockdown of Ape1 sensitized GBM cells to radiotherapy and extended survival in a genetic mouse model of GBM.

### Exosomes

Other siRNA carriers, such as exosomes, have also been studied for siRNA delivery. Exosomes are nano-sized vesicles composed of lipids, proteins, and nucleic acids, with a size of 30–150 nm (Olejarz et al., 2020). Exosomes can penetrate many biological membranes, and the specific proteins and lipids on the membrane facilitate efficient fusion between exosomes and target cells (Luan et al., 2017). In addition, the small size of exosomes can avoid being cleared by mononuclear phagocytes, and has high permeability and retention effect in solid tumor sites (Lu et al., 2018), which can realize the aggregation of drugs at the target site. Since exosomes are biological NPs only composed of biological substances secreted by cells, exosomes are excellent natural carriers for siRNA with low immunogenicity and toxicity compared with other carriers. Exosomes can transmit information between cells and regulate complex biological activities. Due to their low immunogenicity, small size, high enhanced permeability and retention (EPR) and other advantages, exosomes have been widely explored as an alternative to synthetic NPs. Exosomes are widely used in drug delivery, including small molecule chemical drugs, proteins, and nucleic acids (Ha et al., 2016). Moreover, exosomes can enter the blood circulation in the brain through the BBB and thus can be used as delivery vehicles for glioma therapeutics (Fu et al., 2021). For example, the FGFR3-TACC3 fusion gene (F3-T3) has been proven to drive gliomagenesis in GBM (Parker et al., 2013). Parker and colleagues (Parker Kerrigan et al., 2020) demonstrated that targeting F3-T3 using siRNAs specific to the fusion breakpoint could eradicate F3-T3 cancers, and mesenchymal stem cell-derived exosomes were able to successfully deliver iF3T3 *in vivo*, resulting in cell death while avoiding normal tissue toxicity. Recently, Fu and colleagues (Fu et al., 2021) established a new generation of siRNA delivery and gene therapy technology based on the strategy of self-assembled exosomes *in vivo*. They injected mice with synthetic biology gene loops (plasmid DNA) that can express siRNA and exosomal membrane surface targeting peptide to reconstruct the liver of mice into organs that can produce and secrete siRNA, with the attention to realize the stable, efficient, and safe *in vivo* delivery of siRNA through the exosomal transmission pathway. Results showed that this exosomal siRNA could efficiently pass through the BBB and deliver siRNA to the CNS. Furthermore, researchers have successfully implemented target gene interventions for GBM, lung cancer, and obesity through this strategy and achieved significant therapeutic effects. Altogether, this evidence proves that exosomes have significant clinical transformational value for siRNA delivery. However, due to the low yield of exosomes isolated from cells, developing safer and more abundant sources of exosomes is of great significance. In addition, the quick clearance short half-life *in vivo* limits the clinical application of exosomes, which requires further research.

### siRNA-Conjugated Systems

In addition, the conjugation of drugs to siRNA directly has been another promising strategy for targeted delivery. The common conjugate materials are small molecule drugs, peptides, aptamers and lipids (Lee et al., 2016). In line with this, siRNA delivery systems have been developed by directly conjugating siRNA to peptides. For example, researchers synthesized an anti-tumor conjugate comprising methoxy-modified EGFR siRNA and cyclo RGD (cRGD) peptides, which selectively bind to αvβ3 integrins. Results demonstrated that cRGD-siEGFR might be a promising way for glioma treatment, significantly inhibiting tumor growth *in vitro* and *in vivo* with high targeting ability, substantial anti-tumor effects, and low toxicity (He et al., 2017). However, compared with carrier delivery, siRNA conjugates have shortcomings. For one thing, a single delivery carrier can deliver thousands of siRNA molecules simultaneously, while each conjugate has only 1–10 siRNA molecules. For another, the delivery carrier was able to co-delivery siRNA and other anti-tumor drugs.

Zhou (Zhou et al., 2021) and colleagues loaded siRNA surviving into a DNA tetrahedron (TDN). They changed one side of the DNA nanostructure with an aptamer as1411 (As-TDN-R) to enhance the active targeting of these NPs, which effectively downregulated the expression levels of survivin protein and mRNA. Compared with TDN alone, there was increased intercellular uptake of As-TDN-R by U87 cells. The efficiency of siRNA conjugated systems needs to be improved for future clinical application.

## Co-Delivery of Small Interfering RNA and Anti-Cancer Compounds

In the field of glioma therapy, some anti-tumor agents are employed. However, the chemotherapeutic agents are not entirely efficient in cancer eradication. Recently, the combined administration of gene-specific siRNA and chemotherapy or radiotherapy has shown a synergistic effect in glioma treatment. For example, AZD0530 is a dual inhibitor of Src family kinases/Abl, inhibiting Src activation and preventing cell growth by inducing G1/S cell cycle arrest (Nygaard et al., 2015). The combined treatment of LV-STAT3 siRNA and AZD0530 shows better effects in inhibiting the proliferation and inducing the apoptosis of GBM cells than using LV-STAT3 siRNA or AZD0530 alone (Liu et al., 2019). Similarly, Pang and colleagues used self-assembling fluorescent virus-like particle/RNAi nano-complexes to downregulate the hepatocyte growth factor receptor gene, inhibiting the DNA repair mechanism. Furthermore, these complexes can cooperate with TMZ to promote clinical chemotherapy. These findings demonstrated that designing RNAi-based gene therapy can promote chemotherapy for brain tumors (Pang et al., 2019). Additionally, ultrasound irradiation combined with siRNA delivered by nanobubbles might be another ideal approach to achieving glioma’s noninvasive treatment (Cai et al., 2018).

Given the significant advantages of the combined administration of gene-specific siRNA and chemotherapy or radiotherapy, developing co-delivering strategies for siRNA and chemotherapeutics is significant for glioma treatment. Some materials for the co-delivery of siRNA and chemotherapeutic drugs have been developed, and the details are summarized in Table 2. For instance, Lu and colleagues designed a novel heat-sensitive hydrogel to co-deliver anticancer drugs and siRNA. The *in situ* formed hydrogel can prolong drug release and broaden the cancer treatment provided by the chitosan-based copolymer structure (Lu et al., 2020). Similarly, Zhang and colleagues constructed iron oxide NPs with attached carboxyl groups modified to co-deliver siRNA targeting glutathione peroxidase four and cisplatin with high drug loading efficiencies. They then modified the surface of NPs with folate to achieve low systemic toxicity. Results showed that the folate-modified iron oxide NPs exerted substantial effects on U87MG and P3#GBM cells but had limited effects on normal human astrocytes, likely due to higher expression of folate receptor 1 and folate receptor beta in GBM cells. These nanoformulations might represent safe and efficient ferroptosis and apoptosis inducers for combinatorial GBM therapy (Zhang et al., 2020b). In line with this, a polymeric micelle prepared based on a folate-conjugated triblock copolymer of poly (ε-caprolactone) (PCL), PEI, and PEG has been developed to carry TMZ and Bcl-2 siRNA for gliomas treatment (Peng et al., 2018).

**TABLE 2:** Co-delivery of siRNA and anti-cancer compounds.

Carrier	Targeted genes/proteins	Combination	Tumor model (glioma cells)	Results	References
Chitosan hydrogel	SLP2	Irinotecan, cetuximab	Xenograft tumor (U87)	Prolong drug release, and broaden the treatment of cancer.	Lu et al. (2020)
IONPs	GPX4	Cisplatin	Intracranial xenograft (U87MG-luc)	Represent safe and efficient ferroptosis and apoptosis inducers in combinatorial GBM therapy.	Zhang et al. (2020b)
Fa-PEC	BCL-2	Temozolomide	Orthotropic glioma (C6)	Facilitate co-delivery of siRNA and TMZ into C6 cells, resulting in a robust apoptotic response.	Peng et al. (2018)
Ap-CSssSA	VEGF	Paclitaxel	Tumor-bearing mice (U87MG)	Exhibiting great superiority in glioma growth suppression, accompanied by an evident inhibition of neovascularization.	Wen et al. (2020)
Ligand modified NPs	YAP	Paclitaxel	Orthotopic GBM-bearing mice model (U87MG)	Effectively deliver to invasive tumor sites	Yang et al. (2020)
Peptides-modified liposomal	VEGF	Docetaxel	Intracranial GBM models (U87 MG-Luc-GFP, U87)	Deliver siRNA and chemotherapeutic molecules across the BBB and BTB.	Yang et al. (2017)
A2-N	GOLPH3	Gefitinib	Tumor-bearing mice (U87-GFP-Luci)	Successfully crossed the BBB and targeted gliomas, promoting the degradation of EGFR and p-EGFR.	Ye et al. (2019)
NPs	TGF-β	Temozolomide	Tumor-bearing mice (GL261)	Strong siRNA condensation, high drug loading efficiency, efficiently cross the BBB, good serum stability, and magnetic property promotes endosomal/lysosomal escape, enhance the cytotoxicity of TMZ and improve gene silencing efficiency of siRNA	Qiao et al. (2018)
A2PEC	PLK1	Temozolomide	Nude mice glioma model (U87-luci)	Enhance the efficacy of TMZ.	Shi et al. (2020)
Cationic NE	CD73	Temozolomide	Orthotropic glioma (C6)	No synergistic or additive *in vivo* was promoted.	Azambuja et al. (2020a)

Notes: BBB, blood-brain barrier; BTB, blood-tumor barrier; IONPs, iron oxide nanoparticles; Fa-PEC, folate-conjugated triblock copolymer; Ap-CSssSA, angiopep-2 (Ap) modified redox-responsive glycolipid-like copolymer; A2-N, angiogenic protein 2 modified cationic lipid poly (glycolic acid-glycolic acid) nanoparticles; A2PEC, vascular endothelial diphosphate modified polymer micelle; NE, nanoemulsion.

Paclitaxel (PTX) is a natural anticancer drug extracted from plants with poor water solubility. Researchers developed an angiopep-2 (Ap) modified redox-responsive glycolipid-like copolymer co-delivering siVEGF and paclitaxel (PTX). These copolymers are superior in suppressing glioma growth *via* receptor-mediated targeting delivery and cell apoptosis, accompanied by an evident inhibition of neovascularization induced by VEGF (Wen et al., 2020). YAP is a transcription co-activator of the Hippo Pathway and plays an essential role in regulating cell proliferation and apoptosis (Ma et al., 2016). Yang et al. (2020) designed a dual-targeting delivery system based on hepatitis B core protein-virus-like particles with the brain targeting peptide TGN for BBB penetration and RGD ligand for GBM targeting. Paclitaxel and YAP siRNA are packaged together in these NPs. As a result, these carrier packaged agents can be effectively delivered to invasive tumor sites. Moreover, the combination of chemotherapy and siRNA therapy demonstrated synergistic antitumor effects. In addition, docetaxel is obtained by structural modification of PTX (Upadhyay et al., 2010). Yang and colleagues designed a multifunctional liposome system based on anchoring two receptor-specific and penetrable peptides. This dual peptide-modified liposome system can deliver siRNA and chemotherapeutic molecules across BBB and the blood-tumor barrier (BTB) to target GBM (Yang et al., 2017).

Therapies targeting EGFR are promising methods for glioma treatment. Gefitinib (Ge) is an EGFR tyrosine kinase inhibitor (TKI), which cannot enter the brain parenchyma due to the BBB (Ye et al., 2019). Therefore, effective strategies for Ge transporting are needed. Golgi Phosphoprotein 3 (GOLPH3) was reported to promote tumor progression by regulating the transport of cell surface receptors (Zhou et al., 2017). Ye and colleagues developed an angiogenic protein 2 modified cationic lipid poly (glycolic acid-glycolic acid) NPs, to co-delivery Ge and GOLPH3 siRNA. *In vitro* and *in vivo* studies proved that this cationic lipopolymer could successfully cross the BBB and target gliomas (Ye et al., 2019). The released siGOLPH3 effectively silenced the expression of GOLPH3 mRNA and further promoted the degradation of EGFR and p-EGFR (Ye et al., 2019).

Furthermore, studies have confirmed that combining gene therapy with chemotherapy effectively reduces drug resistance and synergistically enhances the anti-cancer effect. Tumor growth factor β (TGF-β) is an immunosuppressive cytokine. Elevated levels of TGF-β in the tumor microenvironment can inhibit the proliferation of T cells and B cells and promote the proliferation of T regulatory cells, thereby reducing the effect of chemotherapy (Ksendzovsky et al., 2009). Qiao and colleagues (Qiao et al., 2018) employed a nano-therapeutic system with dual targeting and ROS response to co-delivery of siTGF-β and TMZ to treat intracranial GBM. These NPs exhibit strong siRNA aggregation, high drug loading efficiency, good serum stability and magnetic properties, which can effectively cross the BBB and target GBM cells through receptor-mediated endocytosis. Furthermore, this nanotherapeutic system contains zwitterionic lipid (distearoyl phosphoethanolamine-polycarboxybetaine lipid). When the nanosystem was internalized into endosomes/lysosomes, the zwitterionic lipid gradually became positively charged as the acidic proceeding. This proceeding led to the perturbation of the membrane of endosomes/lysosomes. It helped TMZ and siTGF-β nano-particles escape into the cytoplasm, promoting endosome/lysosome escape, thereby enhancing the cytotoxicity of TMZ and increasing the gene silent efficiency of siTGF-β. The results show that this nano-therapy system significantly improves the immunosuppressive microenvironment, prolongs the survival time of glioma mice, and can be used to treat brain tumors. Similarly, Bcl-2 and Bcl-xL are anti-apoptotic Bcl-2 family proteins frequently overexpressed in cancer (Adams and Cory, 2007). It was reported that using siRNA to inhibit Bcl-2 and Bcl-xL enhanced the sensitivity of GBM cells to multiple drugs (Halatsch et al., 2019). In line with this, using siRNA to knockdown heat shock protein 27 (Hsp27), one of the marker proteins known to cause GBM treatment resistance, was reported to enhance the therapeutic effect of resveratrol in glioma cells (Önay Uçar and Şengelen, 2019). In addition, the vascular endothelial diphosphate modified polymer micelle was reported to enhance the efficacy of TMZ and the cellular uptake of siPLK1, of which this polymer micelle wraps TMZ through hydrophobic interaction (Shi et al., 2020). siPLK1 is compounded with TMZ-A2PEC through electrostatic interaction (Shi et al., 2020).

In summary, research on siRNA has achieved great success. However, it is essential to improve siRNA’s safety, delivery, pharmacokinetics, and pharmacodynamics to make it a successful therapeutic agent. Thus, the future success of siRNA therapy will depend on the successful development of nano-carriers with better loading, blood transfusion, and safety. Moreover, peptides have multifunctional potential in crossing the BBB and enhancing uptake into the tumor cells. And siRNA encapsulated with peptide-modified NPs can serve as a promising therapeutic avenue for glioma (Wanjale and Kumar, 2017; Zhou et al., 2019). Furthermore, exosomes have significant clinical transformational value for siRNA delivery (Fu et al., 2021). Finally, combination therapy of siRNA and chemotherapy or radiotherapy shows excellent clinical potential, and further preclinical and clinical investigations are needed to verify.

## Conclusion and Prospects

The present review summarized the recent efforts that use siRNA to therapeutically target cancer markers in gliomas, especially the siRNA delivery strategies and their potential in glioma treatment. Once inside the cell, siRNAs from the RISC and subsequently destroy the target mRNA. Recently, CRISPR-Cas9 gene-editing technology has also been developed for gene silencing, and the knockdown efficiency of CRISPR-Cas9 technology seems to be higher than that of RNAi. But there are two major obstacles to using CRISPR-Cas9 technology for cancer treatment: the inefficiency of tumor editing and the potential toxicity of existing delivery systems (Cheng and Wei, 2020; Rosenblum and Gutkin, 2020). Moreover, ethical issues and germline applications of CRISPR-Cas9 need to be considered before translating CRISPR-Cas9 into therapeutic applications.

Compared with other drugs, siRNA drugs show great advantages in treating certain diseases, mainly in the following aspects: 1) siRNAs can target different target genes through sequence design to treat different diseases (Sajid and Moazzam, 2020). For example, for proto-oncogenes or apoptotic genes that play important roles in the process of carcinogenesis, siRNA can be rationally designed according to the sequence of their mRNA to block the expression of these cancer-related genes specifically, thereby inhibiting the growth of cancer cells from treating tumors; 2) siRNAs are easy to design and show efficient silencing effect. 3) siRNA has efficient target specificity. For kinases of the same family, small molecule inhibitors often show off-target effects and side effects. However, using siRNA drugs can minimize off-target generation through precise sequence design; 4) The effect of siRNA is easy to detect. The mechanism of action of siRNA is simple, and the effect of reducing the expression of the target gene is achieved by directly degrading the mRNA of the target gene. Therefore, the effect of siRNA drugs can be detected by qRT-PCR and Western Blot. Small molecule inhibitors usually bind to specific sites of the protein and affect the activity of the protein. Still, the mRNA and protein levels of the target gene are not changed, so it is not easy to detect the effect of small molecule inhibitors. Overall, own to their advantages of abundant targets, convenient synthesis, short preclinical development cycle, and controllable adverse reactions, accompanied by the continuous progress of gene sequencing and targeted delivery technology in recent years, siRNA drugs have attracted more and more attention.

However, siRNAs cannot cross the lipid membrane and directly target patients’ cancer cells due to polyanionic charges and other barriers. For brain tumors, the BBB is an additional obstacle to siRNA delivery. Therefore, suitable delivery strategies are urgent requirements for siRNA-based therapies. Methods to improve the delivery efficiency of siRNA include artificial carriers or coupling ligands. Advancements in nanotechnology research have provided an opportunity to efficiently deliver siRNAs to target cells and increase their cellular uptake *in vivo*. Moreover, combining siRNAs with other therapeutic agents is a beneficial strategy for glioma patients. NPs formed by the mixing and self-assembly of various components are currently an advanced system for siRNA delivery. Still, this formulation method challenges expanding the manufacturing process, such as strictly controlling the mixing process to achieve consistent drug product quality (Gindy et al., 2012). Further research on tumor molecular profiles is also needed to determine the molecules involved in tumor pathogenesis targeted by siRNA and explore new strategies for highly selective and safe siRNA delivery.

Despite creating many nanocarriers with different functionalities being developed in the laboratory, only a few have been approved for clinical use among the abundance of successful pre-clinical studies. The ultimate availability of siRNA for destroying mRNA remains low. Generally, the use of NPs may enhance drug delivery and uptake in glioma. However, less than 1% of an NP-formulated drug dose is found in the brain after systemic injection, and the bulk is delivered into the liver. Thus, there are still challenges in bringing the full potential of siRNA to the clinic. It indicates vast scope for creativity and innovation in the design of siRNA delivery materials. Strategies for endosomal escape, cell and tissue targeting, and developing novel biomaterials are crucial for transforming siRNA from the lab to the clinic. Future efforts for siRNA delivery materials may include: 1) developing new targeting ligands and chemical probes that specifically bind to the surface markers of diseased cell populations, 2) improving the efficiency of siRNA in the cytoplasm; 3) improving the efficiency of siRNA through the BBB; 4) reducing the toxicity to expand the scope of treatment, 5) designing materials with degradable metabolites and 6) simplify preparation procedures.

Finally, we searched for siRNA clinical trials on the ClinicalTrials.gov website. We found that 85 clinical trials have been registered. There are 24 studies for various cancers and 61 studies for other diseases. Four studies focused on solid tumors, and one study included metastatic malignant neoplasm in the brain (NCT03087591). Most clinical trials are in phase 1 or 2, and there is still a long way to go before they are applied in the clinic. Clinical trials have to confirm the effectiveness of siRNA drugs, the delivery efficiency of carriers, and the therapeutic effects of radiotherapy or chemotherapy combined with siRNA drugs. In addition, siRNA drugs have not solved large accumulation in the liver for years, and the targeting efficiency needs to be improved. Therefore, it is necessary to increase research and development intensity further to apply this technology to the clinic fully.
